# Anaplastic Thyroid Carcinoma With Cardiac Dysfunction Developed During Combination Therapy With Dabrafenib and Trametinib

**DOI:** 10.7759/cureus.109105

**Published:** 2026-05-18

**Authors:** Sorane Maezumi, Hideki Tanaka, Yuri Ueda, Takahito Kondo, Kiyoaki Tsukahara

**Affiliations:** 1 Otolaryngology - Head and Neck Surgery, Tokyo Medical University, Tokyo, JPN

**Keywords:** anaplastic thyroid carcinoma, cardiac disorders, dabrafenib, elder patient, trametinib

## Abstract

Anaplastic thyroid carcinoma (ATC) is one of the most aggressive malignancies and is associated with an extremely poor prognosis. Multimodal therapy, combining surgery, radiation therapy, and chemotherapy, is recommended. Although paclitaxel and lenvatinib have been used as drug treatments, combination therapy with dabrafenib and trametinib has recently been reported to be effective. This combination therapy is generally associated with few serious adverse events, although rare cases of cardiac dysfunction have been reported.

Here, we report the case of an 84-year-old woman with* BRAF *V600E mutation-positive ATC who developed cardiac dysfunction during combination therapy with dabrafenib and trametinib. The patient had no significant medical history and was referred to our department with left vocal cord paralysis and a thyroid mass. Histological examination of the right thyroid lobe tumor revealed components of undifferentiated carcinoma. Imaging studies showed invasion of the larynx and trachea, leading to total thyroidectomy, total laryngectomy, and paratracheal lymph node dissection. Postoperative pathological examination confirmed ATC (pT4aN0). Postoperative imaging revealed no distant metastases, and adjuvant radiotherapy was administered. Four months after the completion of radiotherapy, a cervical lymph node recurrence and right lung metastases were observed. As the tumor tested positive for *BRAF* V600E mutation using the MEBGEN™ BRAF kit, combination therapy with dabrafenib and trametinib was initiated. At week five of treatment, pitting edema developed in both lower limbs, and a decrease in the left ventricular ejection fraction (LVEF) was noted, prompting the discontinuation of therapy. By week six, the LVEF recovered, and dabrafenib was restarted at the same dose, whereas trametinib was resumed at a one-step reduced dose. Follow-up CT at week six demonstrated a partial response (PR). Treatment was continued at these doses; however, tumor enlargement was observed at week 26, leading to treatment discontinuation and a transition to palliative care.

ATC remains extremely challenging to treat because of its rapid progression and low sensitivity to drug therapies. Furthermore, lenvatinib, a molecularly targeted agent, shows limited efficacy against ATC and is associated with frequent adverse events. In contrast, combination therapy with dabrafenib and trametinib is considered an effective therapeutic option for patients with *BRAF* V600E mutation-positive ATC, when appropriate management and monitoring are implemented.

## Introduction

Anaplastic thyroid carcinoma (ATC) is a rare histological subtype of thyroid cancers, accounting for approximately 1.3-9.8% of all thyroid cancers [1]. However, the one-year overall survival (1-year OS) rate is only approximately 20%, indicating an extremely poor prognosis [1,2]. Multimodal therapy, which combines surgery, radiation therapy, and chemotherapy, is commonly used to treat ATC. Among chemotherapeutic agents, paclitaxel has traditionally been used, and the molecularly targeted drug lenvatinib has also been approved as a treatment option. Nevertheless, a meta-analysis of 10 studies evaluating lenvatinib reported a partial response (PR) rate of only 15% and a median overall survival (mOS) of 3.16 months, highlighting the limited efficacy of this treatment for ATC [3].

In contrast, combination therapy with the BRAF inhibitor dabrafenib and the MEK inhibitor trametinib demonstrated favorable outcomes in a Phase II trial with an objective response rate (ORR) of 56% [4]. Furthermore, although only five patients with ATC were included, a Phase II trial of another BRAF/MEK inhibitor combination, encorafenib and binimetinib, reported an ORR of 80%, suggesting similar potential efficacy [5]. These findings indicate that BRAF-targeted therapy may be as an effective treatment option for ATC. In Japan, a combination of dabrafenib and trametinib was approved for insurance coverage in November 2023 for unresectable, advanced, or recurrent solid tumors harboring the *BRAF* V600E mutation. However, cardiac dysfunction is a rare adverse event associated with this regimen [6,7].

Here, we report the case of a patient with ATC who developed cardiac dysfunction during combination therapy with dabrafenib and trametinib, but ultimately achieved a favorable clinical outcome. This case was also presented as an abstract at the 2nd Joint Meeting of the Tri-Head and Neck Society held on September 19-20, 2024.

## Case presentation

The patient was an 84-year-old woman. Two months before presentation to our department, the patient developed pharyngeal discomfort and hoarseness and visited her primary care physician. Laryngoscopy revealed no abnormalities, and a follow-up observation policy was adopted. Later that month, swelling developed in her anterior neck, prompting a consultation at another hospital. Cytological examination revealed no malignancy. Two months later, her hoarseness worsened and she revisited her primary physician. Left vocal cord paralysis and a thyroid tumor were detected, leading to a referral to our department for further evaluation. Ultrasound-guided fine-needle aspiration cytology revealed atypical epithelial cells containing undifferentiated components, and MRI demonstrated findings suggestive of invasion of the larynx and trachea (Figure 1). Based on these findings, ATC was suspected. In the same month, we performed total thyroidectomy, total laryngectomy, and bilateral paratracheal lymph node dissection, and created a permanent tracheostoma. Final postoperative pathology confirmed ATC, which was staged as pT4aN0 pEx2 (Figure 2). A postoperative positron emission tomography-CT (PET-CT) scan showed no evidence of distant metastasis (Figure 3). As adjuvant therapy, the patient received radiotherapy alone (60 Gy in 30 fractions).

**Figure 1:**
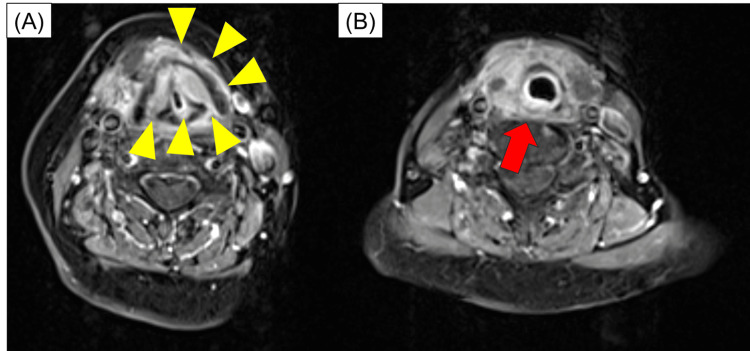
MRI at initial presentation (A) Thyroid tumor invasion of the larynx (yellow arrowheads); (B) Thyroid tumor invasion of the tracheal cartilage (red arrow)

**Figure 2:**
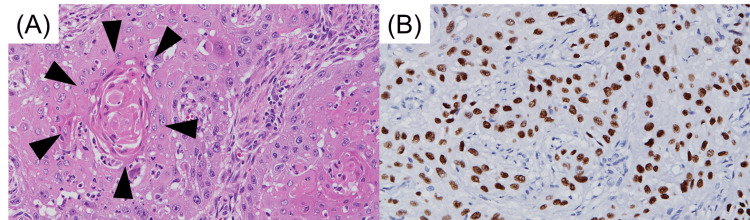
Permanent pathology (A) Keratinization was observed (arrowheads); (B) Positive results for p63

**Figure 3:**
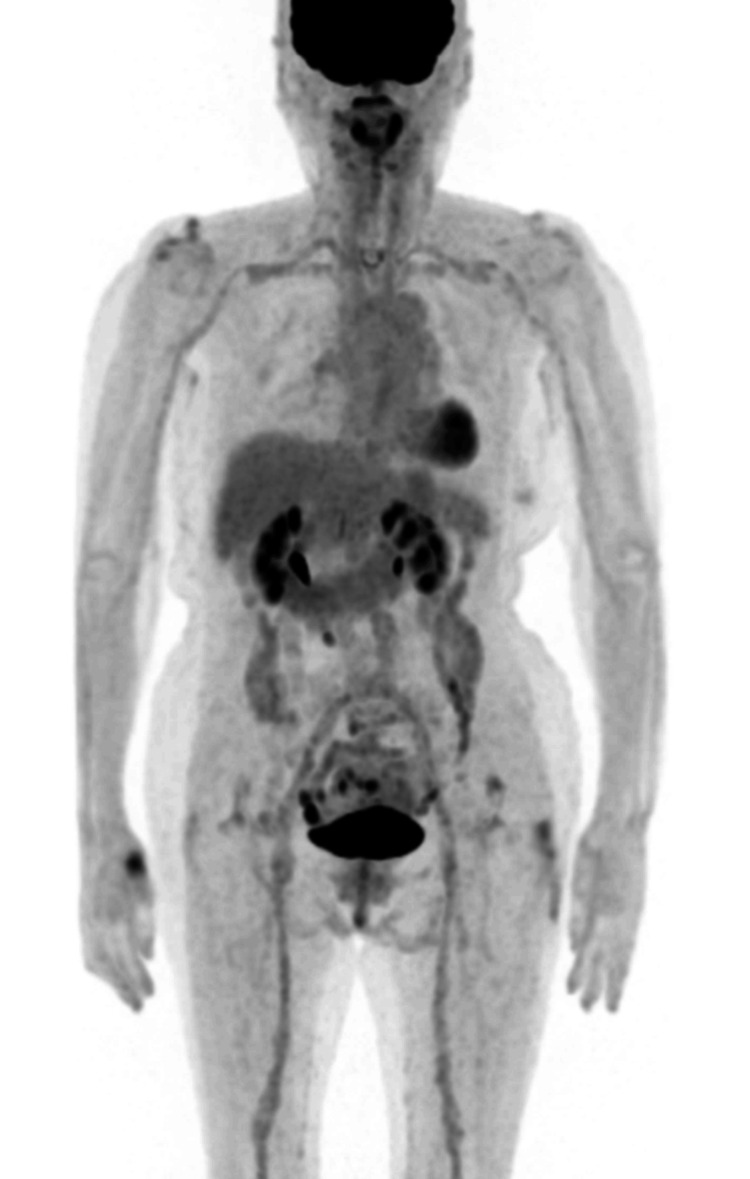
PET-CT after surgery No evidence of distant metastasis. PET: Positron emission technology

During regular follow-up, nine months after surgery, CT revealed metastases in the right supraclavicular lymph node and right lung (Figure 4). CT and MRI revealed metastatic lymph nodes adjacent to the right common carotid and subclavian arteries, with invasion of the skin and trachea (Figures 4, 5). Owing to the proximity of the metastatic nodes to these major vessels, lenvatinib was considered to carry a high risk of bleeding and was not recommended. The MEBGEN™ BRAF kit confirmed a positive *BRAF* V600E mutation, and combination therapy with dabrafenib (300 mg/day) and trametinib (2 mg/day) was administered. Pre-treatment evaluation revealed a left ventricular ejection fraction (LVEF) of 45%. As the LVEF was reduced, the potential risk of cardiac dysfunction was explained to the patient, and combination therapy was initiated. During the first week, Grade 1 nausea (Common Terminology Criteria for Adverse Events (CTCAE) v5.0) developed and was managed with olanzapine (2.5 mg/day) and metoclopramide, resulting in adequate symptom control.

**Figure 4:**
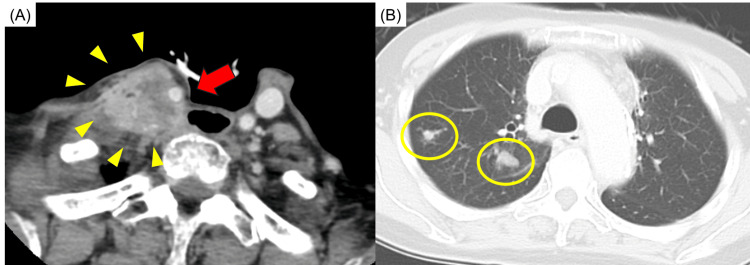
CT at recurrence (A) Right cervical lymph node metastasis (yellow arrowheads) in close proximity to the common carotid artery (red arrow); (B) Pulmonary metastasis (yellow circles)

**Figure 5:**
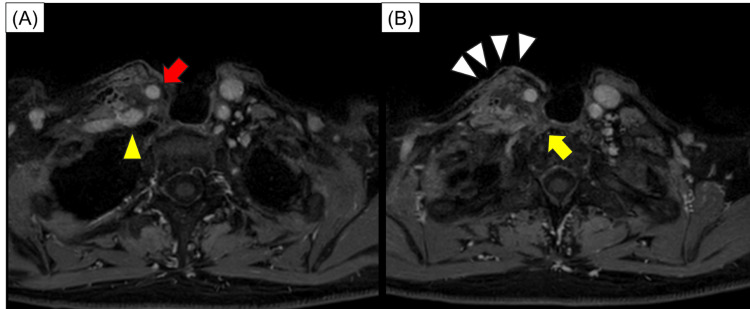
MRI at recurrence (A) Right cervical lymph node metastasis in close proximity to the common carotid artery (red arrow) and the subclavian artery (yellow arrowhead); (B) Right cervical lymph node metastasis with invasion of skin (white arrowheads) and trachea (yellow arrow)

Pitting edema developed in both lower limbs in the fifth week of treatment. Echocardiography revealed an LVEF of 35%, which decreased from baseline. Drug-induced cardiac dysfunction was suspected, and both dabrafenib and trametinib were discontinued. The patient was treated with enalapril maleate, spironolactone, and bisoprolol fumarate was initiated. At the sixth week, the LVEF recovered, and the condition was diagnosed as grade 2 myocardial dysfunction attributed to trametinib. Dabrafenib was resumed at the same dose, whereas trametinib was restarted at a reduced dose of 1.5 mg/day. After resumption, LVEF remained stable at 44%. Follow-up CT imaging at week six showed no significant change in lung metastases but a reduction in the right lymph node, indicating a PR (Figure 6).

**Figure 6:**
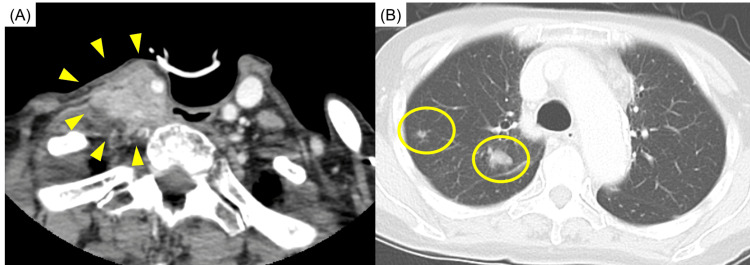
CT at week six of treatment (A) Right lymph node metastasis (yellow arrowheads) was reduced; (B) Pulmonary metastases (yellow circles) were unchanged

At week 26, tumor enlargement was observed, and the treatment was deemed ineffective. Therapy was discontinued, palliative care was initiated, and the patient died 32 weeks after treatment initiation.

## Discussion

In the present case, we administered a combination therapy of dabrafenib and trametinib, which was recently approved for insurance coverage in Japan, to an elderly patient with ATC and achieved favorable clinical outcomes. Although drug-induced cardiac dysfunction developed during treatment, prompt diagnosis and temporary discontinuation of the drugs prevented severe deterioration, allowing the safe resumption of treatment.

The 1-year OS rate for ATC is approximately 20%, and the mOS is five to nine months, reflecting an extremely poor prognosis [1,2]. The treatment typically involves a multidisciplinary approach that combines surgery, chemotherapy, and radiotherapy. Among chemotherapeutic agents, paclitaxel has traditionally been used; in a Japanese prospective study employing weekly administration, the ORR was 21% and the mOS was 6.7 months, indicating limited efficacy [8]. Lenvatinib, a multitarget tyrosine kinase inhibitor with proven efficacy against differentiated thyroid cancer, has shown only limited efficacy against ATC. A Phase II trial in Japan reported a relatively favorable ORR of 24% and an mOS of 10.6 months, leading to its adoption as a therapeutic option for ATC [9]. However, meta-analyses have reported a PR rate of only 15% and an mOS of 3.16 months for lenvatinib monotherapy [3]. Accordingly, lenvatinib is not recommended as a treatment for ATC by the National Comprehensive Cancer Network (NCCN) Guidelines [10]. Moreover, because lenvatinib inhibits vascular endothelial growth factor receptors (VEGFRs), bleeding is a potential adverse event, and fatal cases have been reported [11]. In our case, imaging suggested tumor invasion of the major vessels, trachea, and skin; therefore, the risk of bleeding was considered high, and lenvatinib was deemed unsuitable.

Currently, molecular targeted therapies based on genetic alterations have expanded the treatment options for thyroid cancer. The *BRAF* V600E mutation has been reported in approximately 20-50% of ATC cases [12,13]. BRAF is a mediator of the mitogen-activated protein kinase (MAPK) pathway, and mutations in the *BRAF* gene lead to activation of this pathway, resulting in loss of differentiation, tumor progression, and inhibition of apoptosis [14]. The BRAF inhibitor, vemurafenib, has shown activity against *BRAF* V600E-mutated ATC; however, Phase II trials have reported an ORR of only 29% [15,16]. This limited efficacy may be due to upregulation of HER3 expression, RAS activation, and subsequent MAPK reactivation following BRAF inhibition [17]. In an in vivo study using a mouse model of ATC, a selective BRAF inhibitor provided incomplete inhibition of the MAPK pathway, whereas combination therapy with an MEK inhibitor resulted in complete suppression of this pathway [18]. In the Phase II Rare Oncology Agnostic Research (ROAR) basket trial, combination therapy with the BRAF inhibitor dabrafenib and the MEK inhibitor trametinib demonstrated remarkable efficacy against ATC, achieving an ORR of 56% and a mOS of 14.5 months [4]. In this case, combination therapy with dabrafenib and trametinib was selected because of the confirmed *BRAF* V600E mutation and resulted in a favorable clinical response.

Combination therapy with dabrafenib and trametinib is relatively easy to manage with respect to toxicity. Lenvatinib, although frequently used in thyroid cancer treatment, often causes hypertension and proteinuria, and therefore requires caution in elderly patients, who are more likely to have comorbidities [9]. In contrast, in Phase II trials of dabrafenib plus trametinib, the most common adverse events were fatigue, fever, chills and nausea, and fever was the most common treatment-related serious adverse event [19]. A less common but clinically important adverse event is decreased LVEF. The incidence of LVEF reduction has been reported to be significantly higher with combination therapy using BRAF inhibitors and MEK inhibitors than with BRAF inhibitor monotherapy [6,7]. Trametinib inhibits MEK 1/2, thereby suppressing the downstream activation of ERK 1/2. Because ERK 1/2 is involved in protection against oxidative stress, cardiomyocyte hypertrophy, and apoptosis, its inhibition is considered to potentially induce myocardial dysfunction [20]. According to previous clinical reports, reductions in the ejection fraction have been observed at a mean of 86 days (range: 27-253 days) after treatment initiation [20]. In our case, the patient had no prior history of cardiac disease but exhibited reduced cardiac function before therapy. LVEF declined further at week five of treatment; however, early echocardiographic evaluation and prompt drug withdrawal prevented the progression to severe dysfunction, and cardiac function recovered rapidly. Thereafter, treatment was resumed at a reduced trametinib dose, and stable cardiac function was maintained throughout the subsequent course.

In elderly patients who often have reduced baseline cardiac reserve, cardiac symptoms should be carefully monitored and echocardiography should be performed when clinically indicated. With careful management, combination therapy with dabrafenib and trametinib represents a feasible and effective treatment option for ATC.

## Conclusions

Combination therapy with dabrafenib and trametinib represents a therapeutic option for patients with *BRAF* V600E mutation-positive ATC. Although favorable response rates and median survival have been reported, adverse events including cardiac dysfunction may occur. We present the case of an elderly patient with pre-existing cardiac impairment who was able to continue treatment with appropriate management despite experiencing decreased cardiac function. With careful monitoring and timely intervention, this combination therapy can be considered a feasible and effective option even for elderly patients.
